# Revisiting subclavian flap repair for neonates and small infants

**DOI:** 10.12669/pjms.311.5531

**Published:** 2015

**Authors:** Mustafa Kir, Baran Ugurlu, Nurettin Unal, Kivanç Metin, Nuh Yilmaz, Ozgur Kizilca

**Affiliations:** 1 Mustafa Kir, MD, Department of Pediatric Cardiology, Dokuz Eylul University Faculty of Medicine, Inciralti-Izmir 35340 Turkey.; 2 Baran Ugurlu, MD, Department of Cardiovascular Surgery, Dokuz Eylul University Faculty of Medicine, Inciralti-Izmir 35340 Turkey.; 3 Nurettin Unal, MD, Department of Pediatric Cardiology, Dokuz Eylul University Faculty of Medicine, Inciralti-Izmir 35340 Turkey.; 4 Kivanç Metin, MD, Department of Cardiovascular Surgery, Dokuz Eylul University Faculty of Medicine, Inciralti-Izmir 35340 Turkey.; 5 Nuh Yilmaz, MD, Department of Pediatric Cardiology, Dokuz Eylul University Faculty of Medicine, Inciralti-Izmir 35340 Turkey.; 6 Ozgur Kizilca, MD, Department of Pediatric Cardiology, Dokuz Eylul University Faculty of Medicine, Inciralti-Izmir 35340 Turkey.

**Keywords:** Subclavian flap angioplasty, Aortic coarctation, Children

## Abstract

**Objective:**

We have utilized subclavian flap angioplasty (SFA) frequently in infants with coarctation particularly in patients with arch hypoplasia which is quite frequent. We have followed these patients with serial echocardiography and have analyzed our results in this study to determine recoartation rates, recurrent hypertension and left arm development.

**Methods:**

Thirty eight infants less than 3 months age (22 boys and 16 girls, mean age was 28±22.6 days) operated at Dokuz Eylul University Hospital between August 2007 - December 2013. Twelve (32%) patients with pulmonary banding due to accompanying VSD or AVSD were included to the study, those infants with complex pathologies such as transposition of great arteries or single ventricle, while the patients less than 1000 gram in weight were excluded.

**Results:**

The mean follow-up time was 21 months (1-76 months). Twelve (32%) patients had aortic arch hypoplasia proximal to the left subclavian artery. Operative mortality was found 7.7% for isolated coarctation, 16% for coarctation repair with pulmonary banding. In 5 patients, a residual gradient was detected and re intervention was required in 7.8% patients with balloon angioplasty.

**Conclusion:**

Subclavian flap angioplasty is a safe repair technique in small infants and neonates. High gradients and intervention more likely depends on the anatomy of the aortic arch rather than the subclavian flap angioplasty technique.

## INTRODUCTION

Severly symptomatic Infants with coarctation of the aorta usually require urgent relief of the obstructive lesion. Intervention with balloon angioplasty may provide relief with a relatively simple procedure in small infants but complex lesions with arch hypoplasia can only be treated surgically. Dismal medium and long term results with balloon angioplasty has also led to increased interest in surgical repair in this group of patients.^1^^,^^2^

Currently there are two basic surgical methods for repairing coarctation in small infants; end to end anastomosis (EEA) and subclavian flap angioplasty (SFA) each with its own various modifications.^3^^,^^4^ Traditionally, end to end anastomosis has had a wider following especially with its “extended” variation. A recent analysis of Society of Thoracic Surgeons data base of 5025 patients operated between 2006-2010 has revealed that 89% of patients were treated with a form of EEA while only 3.4% of the patients were treated with subclavian flap angioplasty.^2^

Potential disadvantages of EEA are inadequate growth of the circumferential suture line especially in neonates and small infants. The usual presence of arch hypoplasia or tubular coarctation also entails excessive pulling and tension on the suture lines. Similarly concerns with SFA of, non removal of the ductal tissue with the aortic intima responsible for the coarctation and growth and function concerns regarding the left arm have limited its use.^5^

Long term comparisons regarding recoarctation rates of these two methods have been inconclusive with recoarctation rates varying between 5-30%.^1^^-^^8^ Accumulating evidence suggests that recoarctation is associated more with the complexity of the lesion rather than the type of repair.^8^ Both concerns regarding circular suture lines and residual ductal tissues have proven to be not significant.

Even with declining interest in SFA in most centers we believe SFA still has a role in repairing coarctation in neonates and small infants. Especially infants with arch hypoplasia distal to the left subclavian artery, which is encountered more often than not, may benefit from SFA. With SFA the long segment coarctaiton can be easily repaired with the particularly well developed subclavian artery.^5^ We have analyzed our experience with SFA in neonates and small infants and have followed up these patients with echocardiography to determine recoartation rates, recurrent hypertension and left arm development.

## METHODS

We have retrospectively analyzed data of 38 patients aged less than three months age operated by pediatric cardiac surgeons for aortic coarctation with SFA at Dokuz Eylul University Hospital between August 2007 - December 2013. During this period SFA was the preferred surgical technique in infants particularly for patients with arch hypoplasia distal to the left subclavian artery. Patients with significant arch hypoplasia proximal to the left subclavian artery were treated with extended arch repair and end to end anastomosis and were excluded from the series. Patients with ventricular septal defect or atrioventricular septal defect who underwent pulmonary artery banding (PAB) concomitantly were included but patients with complex congenital cardiac problems such as transposition of the great arteries or single ventricle type lesions and patients weighing less than 1000 grams were not included in the series.

***Preoperative data:*** There were 22 boys and 16 girls, mean age was 28±22.6 days (1-83 days), mean weight was 3.4±0.9kg. 24 (63%) of the patients were neonates of less than 28 days age 16 patients (42%) had a VSD and 12 patients (32%) had a PAB in addition to SFA. All patients were operated for varying degrees of heart failure with 22 patients (58%) on positive inotropes at the time of the operation and 14 patients (37%) had been entubated and on positive pressure ventilation. Balloon angioplasty was performed unsuccessfully in three patients before surgery.

**Fig.1:**
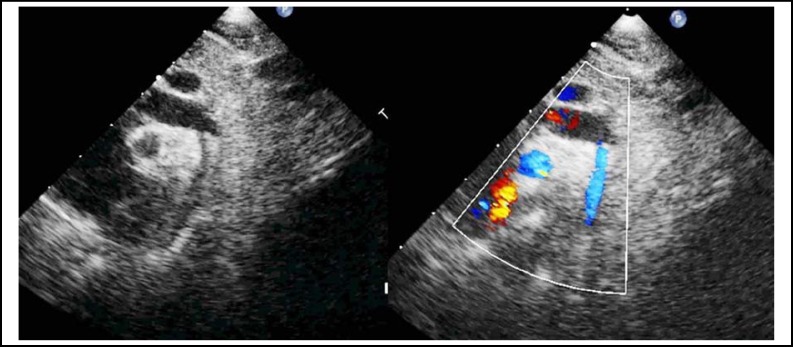
A two-dimensional and color Doppler echocardiographic image obtained from 14 days neonate

***Preoperative echocardiography:*** Arch measurements were made before surgery in all the patients. Mean arch diameter proximal to the left subclavian artery was 6.54±1.13 with 12 (32%) patients having arch measurement less than -2SD Z score (Fig.1). Tubular narrowing distal to the subclavian artery was present in 27 (%) patients with a mean diameter of 4.07±1.18.

**Fig.2:**
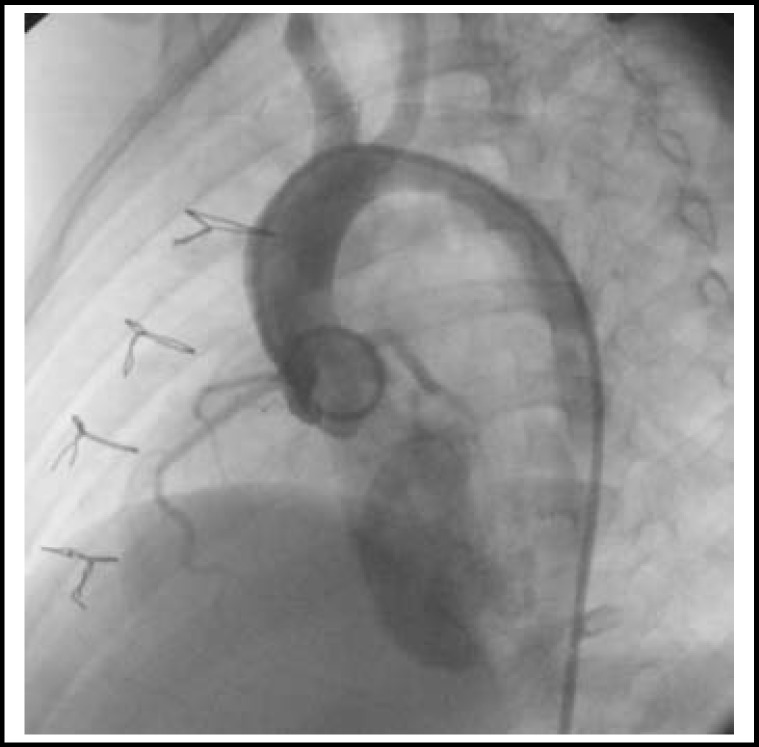
An angiographic view of a patient whose coarctation was removed surgically by the procedure of subclavian flap angioplasty

***Surgical technique:*** All patients underwent surgery through a left thoracotomy. Left subclavian artery was divided distal to the left vertebral artery and distal aortic arch was repaired with the subsequent subclavian flap. The coarctated segment of the aorta was opened longitudinally and the subclavian artery flap was turned downwards to the distal aortic arch and was sutured with continuous 7/0 prolene sutures. Ductus was ligated in all of the operations. In 29 patients (67%) ductal and intimal tissue was removed from inside of the coarctated segment during the repair (Fig.2).

***Statistical analysis:*** Statistical analysis was performed with SPSS 15.0 for windows program. Continuous variables were given as means with standard deviation and median. Significance for continous variables was determined with Mann-Whitney U test for independent samples.

## RESULTS

Four patients died after the repair with an operative mortality of 10.5%. Of the four patients that died two patients had an isolated coarctation repair and the other two patients had an additional PAB. Operative mortality for isolated coarctation repair was 7.7%. Operative mortality for coarctation repair and PAB was 16%. One patient with isolated coarctation was 22 days old and was admitted entubated with severe left ventricle failure; he died 8 days after the operation with multiorgan failure. The other patient was 55 days old and died 2 months after the operation with irreversible neurologic problem, due to intracerebral hemorhage. The patients with SFA and PAB were 13 and 60 days old and both were entubated with heart failure before the operation. One patient was operated in emergency in cardiogenic shock after a failed balloon angioplasty. Median age of the patients that died did not show a significant difference when compared to patients that survived the operation, similarly median weight also did not show a difference (p>0.05).

Prolonged entubation was frequent after surgery with 16 patients (37%) having entubation periods longer than 7 days with 9 of these patients being patients with additional PAB. Correspondingly, patients had long ICU stays with a mean of 9±7days. Chylothorax was encountered in one patient which resolved spontaneously, no serious vocal chord problems were noted.

***Follow-up: ***The surviving patients were followed for a mean period of 21 months (1-76 months, median 10.4 months) All surviving patients received at least one echocardiographic examination postoperatively with a mean number of 4.7±2.6 examinations. Four patients had MRI exams and 3 patients had angiograms after surgery Eventually 4 patients were lost to follow-up with follow-up complete in 89% of the patients while the rest of the patients were controlled within 6 months of the study. 

During the follow-up period 3 of the 4 patients with VSD’s and no banding underwent VSD closure at 2.6 and 7 months after the coarctation repair. Of the 12 patients with PAB 5 patients underwent debanding-VSD closure procedure mean 15.2±3 months after the initial operation. One patient underwent ASD closure 28 months after the coarctation repair.

Follow-up echocardiogram were analyzed for peak gradients. The latest echocardiograms showed a median peak gradient of 13.5±11mmHg in the surviving patients. Five patients (15%) showed a gradient higher than 25 mmHg. Three of these patients had gradients of 40mmHg and 55 and 75mmHg. All 3 of these patients underwent recatheterization and balloon dilatation. The rate of reintervention for coarctation was 8% of the total surviving 34 patients. The remaining 2 patients are currently being followed. Patients that underwent reintervention did not show a significant difference as regards age or weight at operation time (p>0.05). Proximal segments of the subclavian arteries were less than -2SD Z score in all 5 of these patients. Baloon angioplasties were performed for proximal stenotic lesions in all 3 patients. 

In 28 patients, blood pressure measurements from the right arm of the patients at follow ups showed systolic pressures within normal limits. The blood pressure of 5 patients remained over 95 percentile.

Left arm development was inspected visually and arm length was measured at each follow-up. Right arm and left arm measurements and development did not show a difference in all followed patients.

## DISCUSSION

Subclavian flap angioplasty was first described by Waldhausen in 1966 with the premise of providing a living flap for enlarging the coarctated segment of the aorta. The potential problem of recoarctation in neonates and small infants from the circular anastomosis lines of the resection and end to end anastomosis technique prompted an interest in SFA. Subsequent experience showed a recoarctation rate of 5-20% in these patients which was independent of the technique used.^3^^,^^7^ Concerns over the native coarctation tissue left in-situ and left arm development led to a decrease in the use of SFA especially in North America.^2^

From the surgical standpoint SFA has desirable aspects which kept it in use in certain centers despite concerns. It is technically an easy operation with tension free suture lines which is very important in neonates as tissues tend to tear very easily. The subclavian artery is usually well developed in these infants and the flap provides more than adequate enlargement of the coarctated segment. Another overlooked advantage of SFA is the presence of a long hypoplastic segment distal to the subclavian artery. In our experience this proved to be quiete often in this subgroup of patients. The SFA technique easily corrects this problem without the use of extended resection and arch anastomosis. 

We have used SFA quite liberally in this subgroup of patients and 60% of neonates and small infants presenting with coarctation have underwent a SFA procedure. Our operative mortality of 7.7% for isolated coarctation and 10.5% for SFA and banding is high compared to some reported series. But it should be kept in mind that half of the patients were operated with significant left ventricle dysfunction and 1/3 of the patients were already on mechanical ventilation before surgery. SFA could be performed rapidly and effectively in all of the patients. We believe this mortality is related more closely to preoperative patient status rather than operative technique. PAB is an independent risk for mortality which is also apparent in our patient series with 16% mortality for the combined procedure. A single stage repair of coarctation and closure of VSD is an attractive choice in these patient but complex coarctations with tubular hypoplasia usually require circulatory arrest or selective cerebral perfusion.^9^^-^^11^ We have left the VSD open in 4 cases and have observed that in 2 cases the VSDs have closed significantly during the follow-up period. The other 2 patients underwent VSD closure within a few months without a problem. We believe VSDs that do not compromise the patient hemodynamically can be left open without PAB.

The rate of intervention for recoarctation was 8% in our series and compares well with comparable patient series of both end to end repair and SFA. A review of almost 400 patients with SFA followed for 14 years showed a reintervention rate of 15%.^5^ The median follow-up period in our case series is only one year but this could be accepted as adequate as most reinterventions occur within the first year of the coarctation repair. It should also be noted that reinterventions was performed at residual arch lesions proximal to the repair. None of the reinterventions were performed for actual recoartaion at the repair site. We believe residual ductal and intimal tissue with the SFA has proven to be an insignificant factor for recoarctation. The common practice of removing some of the intimal tissue during the repair may also help as regards this potential problem.

The residual gradients in the patients followed-up proved to be insignificant in great majority of the patients with all the patients being normotensive. Control echocardiograms of the patients showed satisfactory aortic growth at the coarctation site. Arch stenosis proximal to the repair also showed a tendency to grow but these problems were also responsible for reinterventions and residual gradients. Left arm development is seems to be effected less than anticipated. In contrast to problems associated with classic Blalock Taussig shunt which requires ligation of all branches of the subclavian artery reducing collateral flow, SFA can be usually performed with ligation of only the vertebral artery. Our patients did not show a difference in growth between the two arms. Studies with longer follow periods into adulthood have shown no significant left arm impairment with this technique.^5^^,^^12^

We believe SFA does have a role in the treatment of neonates and small infants with coarctation. It provides satisfactory relief of the coarctated segment and of the arch hypoplasia distal to the left subclavian artery. Residual gradients and reinterventions are usually more related to the extent of arch problems rather than technique of the operation.

## Authors Contribution:

**MK, BU** conceived, designed and did statistical analysis & wrote the manuscript.

**NU, KM** edited the manuscript, reviewed and gave final approval of the manuscript.

**NH, OK **did data collection.

**MK** takes responsibility and is accountable for all aspects of the work in ensuring that questions related to the accuracy or integrity of any part of the work are appropriately investigated and resolved.
